# Time Related Changes of Mineral and Collagen and Their Roles in Cortical Bone Mechanics of Ovariectomized Rabbits

**DOI:** 10.1371/journal.pone.0127973

**Published:** 2015-06-05

**Authors:** Xin-Xin Wen, Fa-Qi Wang, Chao Xu, Zi-Xiang Wu, Yang Zhang, Ya-Fei Feng, Ya-Bo Yan, Wei Lei

**Affiliations:** Department of Orthopedics, Xijing Hospital, Fourth Military Medical University, Xi’an, China; Rensselaer Polytechnic Institute, UNITED STATES

## Abstract

As cortical bone has a hierarchical structure, the macroscopic bone strength may be affected by the alterations of mineral crystal and collagen, which are main components of cortical bone. Limited studies focused on the time related alterations of these two components in osteoporosis, and their contributions to bone mechanics at tissue level and whole-bone level. Therefore, the purpose of this study was to elucidate the time related changes of mineral and collagen in cortical bone of ovariectomized (OVX) rabbits, and to relate these changes to cortical bone nanomechanics and macromechanics. 40 Rabbits (7-month-old) were randomly allocated into two groups (OVX and sham). OVX group received bilateral ovariectomy operation. Sham group received sham-OVX operation. Cortical bone quality of five rabbits in each group were assessed by DXA, μCT, nanoindentation, Fourier transform infrared (FTIR) spectroscopy and biomechanical tests (3-point bending of femoral midshaft) at pre-OVX, 4, 6, and 8 weeks after OVX. As time increased from pre-OVX to 8 weeks, the mineral to matrix ratio decreased with time, while both collagen crosslink ratio and crystallinity increased with time in OVX group. Elastic modulus and hardness measured by nanoindentation, whole-bone strength measured by biomechanical tests all decreased in OVX group with time. Bone material properties measured by FTIR correlated well with nano or whole-bone level mechanics. However, bone mineral density (BMD), structure, tissue-level and whole-bone mechanical properties did not change with age in sham group. Our study demonstrated that OVX could affect the tissue-level mechanics and bone strength of cortical bone. And this influence was attributed to the time related alterations of mineral and collagen properties, which may help us to design earlier interventions and more effective treatment strategies on osteoporosis.

## Introduction

Osteoporosis is defined by progressive loss of bone mass and impairment of bone quality lead to a reduction in bone strength[1]. Vertebrae and femoral neck are the main sites where osteoporotic fractures happen[2]. As trabecular bone is a main contributor to bone strength in vertebrae and femoral neck, many studies have focused on trabecular bone quality. However, cortical bone is also a significant contributor to whole bone strength, especially for the elderly[3].

Cortical bone has a hierarchical structure, spanning from the macrostructure at several millimeters or whole-bone level, the microstructure at several hundred micrometers level, to the nanostructure at hydroxyapatite crystals and collagen fibrils level[4]. The main components of cortical bone are mineral and collagen. Mineral accounts for nearly 2/3 of the dry weight of bone matrix, and collagen accounts for 1/3 of the dry weight of bone[5]. The macroscopic mechanical properties are closely related with the hierarchical tissue properties of cortical bone, and the whole bone strength may be affected by the alterations at mineral and collagen levels[4, 6].

FTIR uses spectrometers to measure properties of the bone mineral and collagen[7], and it has been used to characterize the bone compositional properties in several studies. The measured parameters that may be related to bone quality are mineral content, mineral crystallinity and collagen cross-link ratio[8]. This technique could be used to characterize the changes of mineral and collagen in osteoporosis and to reveal whether crystal size and collagen maturity are predictive of bone strength. In a recent report analyzing iliac crest biopsies from patients, the author highlighted a 14% higher collagen cross-link ratio in the nonosteoporotic t-scores subjects with low-energy fractures than the ratio in the non-fracture controls[9]. Such fractures in the non-low-BMD subjects could not be attributed to abnormal bone structure or microarchitecture. Higher collagen cross-link ratio was also detected in bone from patients with osteoporosis or high risk for fracture than healthy or non-fracture controls in other former studies[10–12]. The significant differences of mineral properties between osteoporotic bone and control were also demonstrated in former studies[13].

Unfortunately, using human bone biopsies, the time related changes of these properties cannot be easily observed as the duration of estrogen withdrawal increased. Other limitations of these studies were the small amount of tissue available in the biopsies, and the analyzed sites were limited to iliac crest. So, the time-related changes of mineral and collagen in cortical bone tissue as the duration of estrogen withdrawal increased, and how mineral and collagen affected the nanomechanics and whole-bone strength needed to be elucidated carefully.

Rabbits are the species known to have quite fast Haversian bone remodeling processes. In our unpublished study on vertebral cancellous bone, significant decline of BMD in vertebral bodies were detected at 8 weeks after OVX. And μCT analysis showed that the microarchitecture of vertebral bodies were also deteriorative. The lumbar BMD and microarchitecture in OVX group changed significantly relative to sham group at 8 weeks in another study[14]. It demonstrated that osteoporotic model in rabbits was built up successfully at 8 weeks after OVX surgery.

To address these questions, this study aimed to characterize the time related changes of mineral and collagen properties in OVX rabbit cortical bone, and to relate them with nanomechanics and whole bone strength. BMD and structure were also measured. We hypothesized that the changes of mineral and collagen properties became more pronounced as the duration of estrogen withdrawal increased, and those properties made contributions to the decreasing bong strength in nano and whole-bone level.

## Materials and Methods

### 1. Animals

40 female, 7-month-old (3.16±0.27 kg), skeletally mature, New Zealand white rabbits were included in the present study (Fourth Military Medical University, Xi’an, Shaanxi, China). These experiments were approved by the local ethics committee(Fourth Military Medical University Ethics Committee) and the guidelines for care and use of animals were followed. The rabbits were kept individually in cages and maintained on a 12-hr light/12-hr dark cycle at room temperature with ad libitum access to water and standard commercial rabbit chow. The rabbits received general anesthesia in OVX surgery by intramuscular combined administration of pentobarbital and Sumianxin II(Changchun Veterinary Institute of Military Medical Academy of Sciences, China).

### 2. OVX rabbits

After the rabbits were acclimatized to the new situation for 2 weeks, they were randomly allocated into a sham operation group (sham group, n = 20) and an OVX group (OVX group, n = 20). 5 rabbits in each group were euthanized to acquire baseline data(BMD, μCT, nanoindentation, FTIR, and biomechanical test) with no treatment before OVX surgery. Rabbits in group OVX received bilateral ovariectomy in general anesthesia. For the sham-OVX operation on the sham group, the ovaries were held up and then returned to their original positions. Antibiotics (ampicillin, 0.1 g/kg/day, China) were administered before surgery and for 5 days post-surgery to prevent infection. The rabbits were euthanized by intravenous administration of an overdose of Sumianxin II at each time point (4 weeks, 6 weeks, and 8 weeks after operation) in each group. After death, the femora of both sides were dissected, soaked in 0.9% saline solution gauze, and frozen at -80°C in plastic bags for the further examinations.

### 3. DXA

**Fig 1:**
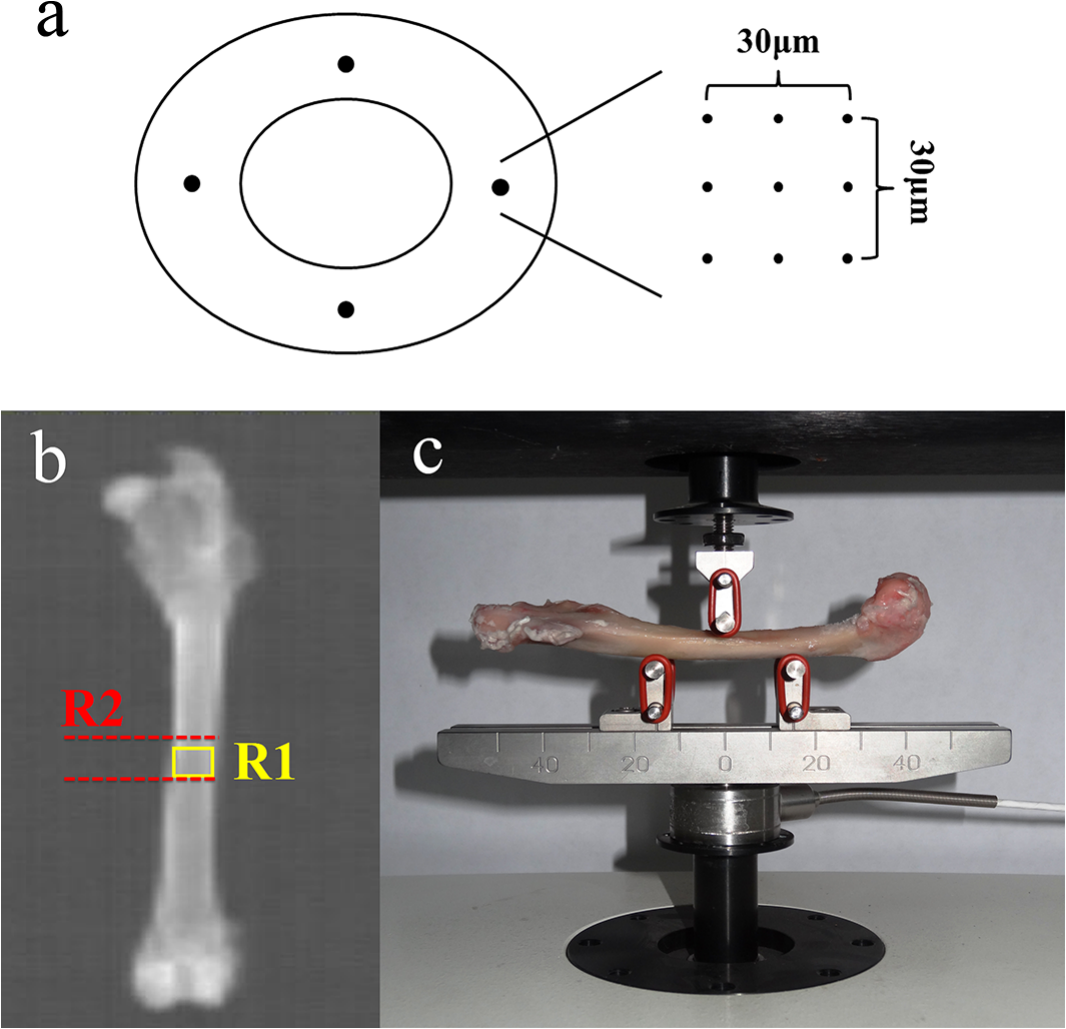
Nanoindentation, DXA and 3-point bending test measurements of femora. (a)Nanoindentation tests were performed in the circumferential direction of the cortical bone in pre-determined grid of 3×3 points at four locations(medial, lateral, anterior and posterior), and the distance between each measurement point in the grid was 10 μm. (b)DXA measurement of femora(8 mm ×12 mm ROI, 4 cm from the femoral distal end, as shown in yellow rectangle R1); 1-cm-long cortical bone specimen R2 used in further analysis was shown between two red dotted lines. (c)3-point bending test measurement of femora.

DXA analysis was performed with a Hologic Discovery Wi using linear fan beam technology (analysis software version 12.7; Hologic Inc., Bedford, MA, USA), switching between two X-ray potentials (100 and 140 kVp) from an X-ray source mounted beneath the subject. The small animal-scanning mode was applied for all scanning. After the rabbits were euthanized, the left femora were underwent BMD scans in vitro. The cortical bone-rich scanning regions of interest(ROI) was at the midshaft of left femora(8 mm × 12 mm, bottom margin of ROI was 4 cm from the femoral distal end, as shown in yellow rectangle R1, Fig 1B) to represent the BMD of cortical bone[15].

### 4. μCT analysis

Bones harvested were frozen at -80°C until the time of scanning by μCT (Healthcare Explore Locus, GE Medical Systems, Milwaukee, USA). The specimens were thawed at room temperature before μCT scan and kept moist by saline during all scanning procedures. A 1-cm-long cortical bone was cut from the right femoral midshaft, which was 4 cm from femoral distal end. This 1-cm-long cortical bone specimen was between two red dotted lines as shown in Fig 1B. The images were obtained using the following parameters: (a) 80 kV as the X-ray tube voltage; (b) 80 μA as the anode current; (c) 3000 ms as the shutter speed; (d) 2 as the binning factor; and (e) 0.5° as angle of increment. The μCT system was used at a spatial nominal resolution of 14 μm, and CT images were reconstructed in 1024×1024-pixel matrices. Microview v2.1.2 software was used. The whole cortical bone specimen was selected as the ROI. The cortical thickness, total cross-sectional area inside the periosteal envelope, cortical area, and cortical area fraction were determined[16].

### 5. Nanoindentation

The 1-cm-long cortical bone specimens that had been scanned by μCT were used for nanoindentation test. The bone tissues were kept frozen at -80°C until the beginning of the specimen preparation and between the procedure steps. The bone marrow were removed by a water jet (Teledyne Water Pit, Fort Collins, CO, USA) and an ultrasonic bath. All the specimens were dehydrated in a series of alcohols to avoid problems that can be caused by surface liquid films in identifying the point of first contact during indenter approach to the specimen surface. After dehydration, the specimens were embedded in epoxy resin. After the embedding process, the specimens were cut transversally through the middle to create completely exposed surfaces. Then the specimens were metallographically polished to produce the smooth surfaces needed for nanoindentation testing, first with silicon carbide abrasive papers (Starcke GmbH & Co. KG, Melle, Germany) of decreasing grit size (600, 800 and 1200 grit), and finally with diamond suspensions (0.3 and 0.05μm particle size) embedded in soft polishing cloths (Buehler, Lake Bluff, IL). The specimens were washed in deionized water between each polishing step to remove debris, taking care that the surface of the bone was not demineralized. The nanoindentation was performed using a TriboIndenter (Hysitron TI90, Minneapolis, MN) with a standard Berkovich tip. The procedure was defined as follows: after the identification of the surface, the tip was loaded into the sample at a rate of 50 μN/s, held at a maximum load of 1000 μN for 10 s, and unloaded at 50 μN/s. After removing 85% of the maximal load, constant load was held for 100 s to measure drifting of the displacement transducer due to both the thermal effect on the transducers capacitor and the viscous effect of the bone tissue behavior. Indentations were performed in a predetermined grid of 3×3 points (10 μm spacing) at four locations (medial, lateral, anterior and posterior) per sample(Fig 1A)[17]. The indentation sites on the cortical bone were placed on the interstitial lamellar bone. Hence, 36 indentations were made in each feature. Bone tissue elastic modulus and hardness were calculated from the unloading segment of the load-displacement curve according to the method of Oliver and Pharr[18]. Mean values for the elastic modulus and the hardness of bone tissue were calculated for each specimen.

### 6. Fourier transform infrared microspectroscopy

The epoxy resin-embedded specimens that were used in nanoindentation test were further analyzed with FTIR microspectroscopy. The embedded tissues were cut transversely at 4 μm thickness using a Polycut E microtome(Jung, Heidelberg, Germany) and mounted on barium fluoride infrared windows for FTIR microspectroscopic analysis (Shimadzu AIM-8800, Shimadzu Corporation, Tokyo, Japan). Each spectrum was acquired with a spatial resolution of 6.25 μm, spectral resolution of 4 cm^−1^ and 16 repeated scans in the wavenumber region between 4000 and 800 cm^−1^. Area measurements, approximately 100 μm × 100 μm each, were performed on the cortical bone at the same locations as the nanoindentation[17]. Contributions of water vapor and epoxy resin were subtracted from the original spectrum. The amide I and II spectral regions were baseline corrected according to the standards published elsewhere[19]. Spectra were curve fitted using a commercially available software package (Grams/32; Galactic Software, Inc., Salem, NH, USA). The initial position and type(Gaussian) of underlying bands that were input were determined through second derivative and difference spectroscopy. Once the curve-fitting process converged, the output of the analysis was expressed as peak position and relative percent area. Parameters calculated were: 1) mineral to matrix ratio, calculated as the ratio of peak areas of phosphate(1200-900 cm^−1^) and amide I(1720-1585 cm^−1^)[20]; 2) crystallinity, related to the size and perfection of hydroxyapatite crystal, calculated as the ratio of relative peak height subbands at 1030 and 1020 cm^−1^[21]; and 3) maturity of the collagen cross-link, calculated as the intensity ratio of amide I subbands at 1660 and 1690 cm^−1^[22]. All the FTIR parameters above were averaged among collected spectra from per rabbit.

### 7. Biomechanical tests

The left femora were tested in 3-point bending tests with the materials-testing machine (MTS 858 System Inc., MN, USA) to assess whole-bone mechanical properties, and to estimate the cortical bone mechanical properties[23]. The specimens were thawed before mechanical tests and kept moist during all handling and test procedures. Femora were tested in 3-point bending tests with the anterior surface on the lower supports (30 mm apart) and the load applied to the posterior surface centered between the lower supports(Fig 1C)[24]. Load was applied perpendicularly to the long axis on the bone at a constant rate of 1 mm/min until fracture. After 3-point bending test, the load-displacement curve was depicted. These parameters can be normalized after adjusting for the sample size (cross-sectional area or moment of inertia), allowing load conversion to stress and deformation to strain, and obtaining the stress-strain curve[24]. The calculating processes were made under the assumptions that the femoral cross sections were elliptically shape(outer and inner contour of the elliptic cortical bone ring with hollow structure was elliptically shape), the cross section was constant, the cortical bone was linear elastic, homogenous, and isotropic. As bones were tested in anterior-posterior bending, we determined that the major axis of the ellipse was the width of the cross section in the medial-lateral direction, and the minor axis of the ellipse was the width of the cross section in the anterior-posterior direction[23, 24]. Young’s modulus, ultimate stress, yield stress, and toughness were calculated using standard engineering formula from the stress-strain curve to assess the cortical bone strength[23, 24].

### 8. Statistical analysis

All data were processed with the statistical system SigmaPlot 12.5 (Systat Software, Inc., San Jose, California, USA) and expressed as mean ± SD. All data were normally distributed. Differences in BMD, structural parameters, nanomechanical, FTIR, and macromechanical parameters between two groups at the same time point were compared with Independent-Samples t Test. Differences of those parameters among different time points in the same group were performed with the one-way ANOVA test. If the one-way ANOVA of one parameter was significant, all pairwise multiple comparison procedures (Student-Newman-Keuls method) were performed in the same group. Linear regressions were performed within sham group or OVX group separately to examine relationships among BMD, microstructure, nanomechanical, FTIR, and macromechanical parameters above. P<0.05 was considered statistically significant.

## Results

### 1. DXA

**Fig 2:**
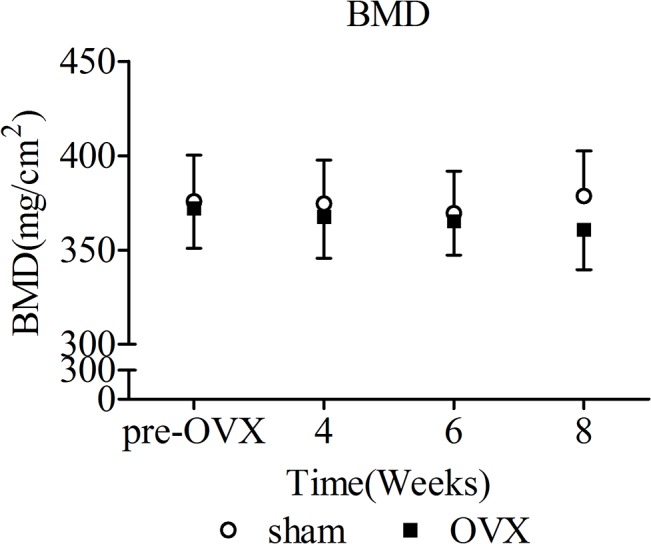
BMD (mg/cm^2^) values(mean ± SD) in femoral midshaft. No significant differences were detected between OVX and sham group.

BMD values of femoral midshaft were shown in Fig 2. There were no significant differences of BMD values between OVX and sham group at pre-OVX, 4, 6, and 8 weeks. No temporal changes of BMD in femoral midshaft were detected in sham group or OVX group. OVX operation did not have an influence on apparent bone mineral density of femoral midshaft.

### 2. μCT

**Fig 3:**
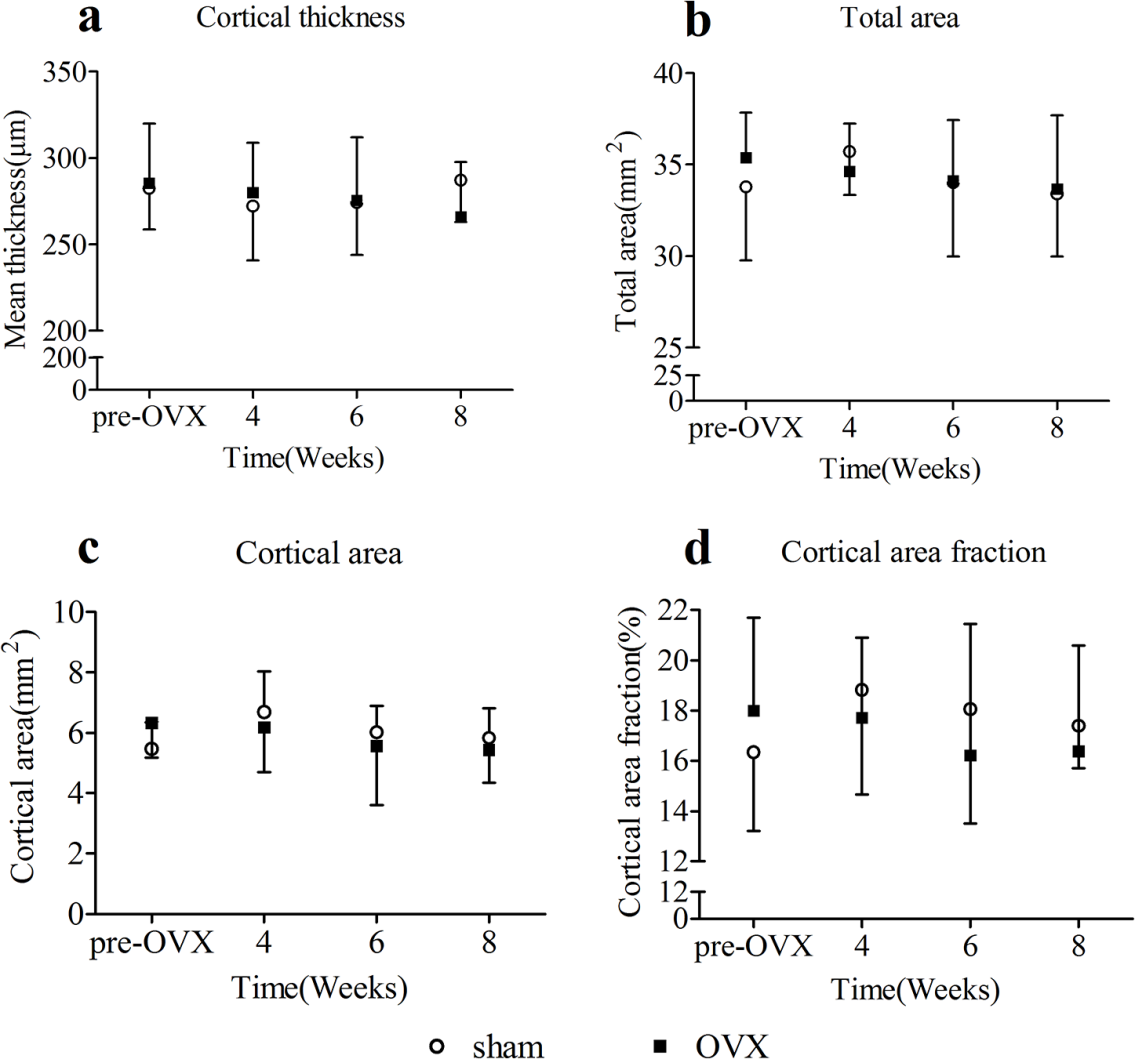
Overview of the μCT analysis results of femoral cortical bone(mean ± SD). No significant differences of cortical thickness(a), total area(b), cortical area(c), and cortical area fraction(d) were detected.

The structural properties of femoral cortical bone were shown in Fig 3. The structural properties of femoral cortical bones, consisted of cortical thickness, total area, cortical area, and cortical area fraction, in OVX group did not show any significant difference against sham group at the same time point during the experiment. No temporal changes of structural properties of cortical bones were detected in OVX group or sham group. OVX operation did not have influences on the structure of cortical bone.

### 3. Nanoindentation

**Fig 4:**
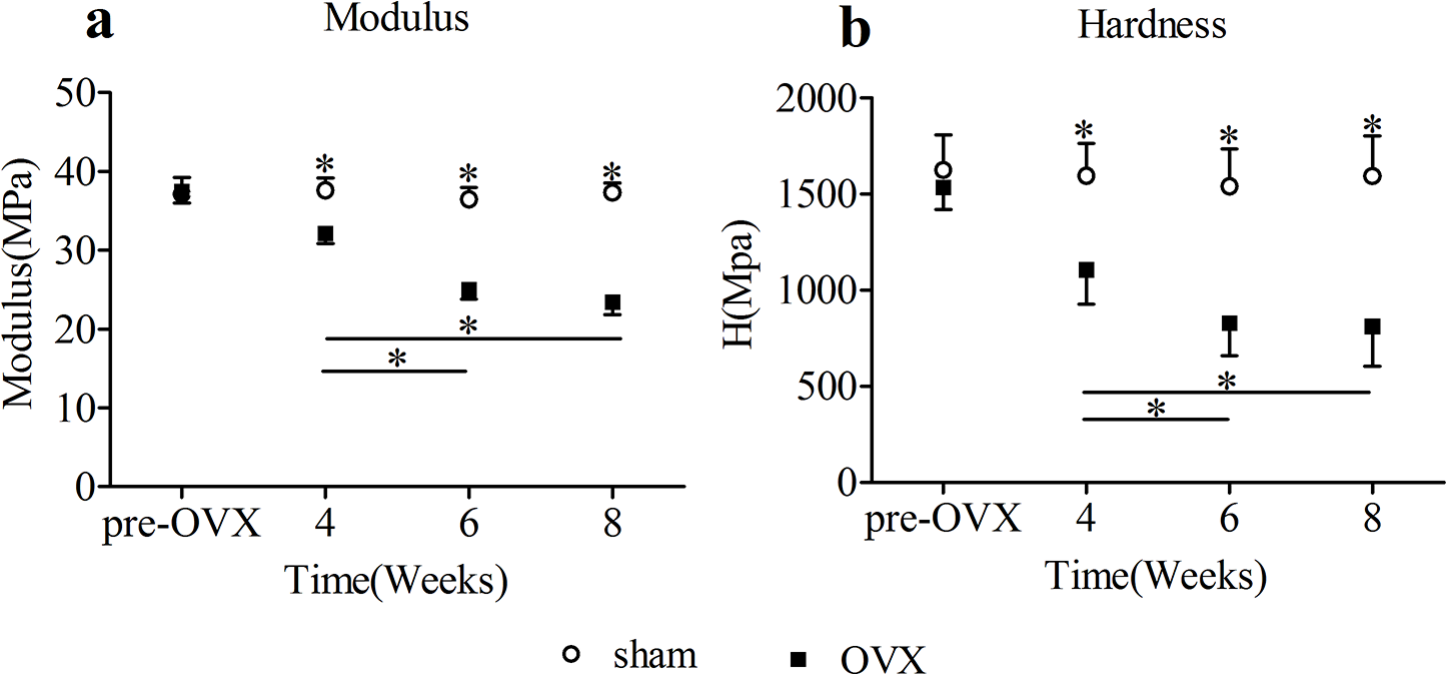
Nanoindentation results(mean ± SD) of femoral cortical bone specimens. Asterisks showed the statistically significant difference between OVX group and sham group at the same time point. The values of modulus(a) and hardness(b) in the OVX group decreased significantly from 4 weeks on. Transverse lines with asterisks above showed the differences in OVX group among different time points. *p < 0.05.

Results of nanoindentation tests on femoral cortical bone specimens were shown in Fig 4. 4 weeks onwards the values of elastic modulus and hardness of cortical bone in OVX group decreased significantly relative to sham group. As the time increased from pre-OVX to 8 weeks, the elastic modulus and hardness both decreased. Nanomechanical parameters of femoral cortical bone specimens in OVX group changed relative to sham group at 4 weeks(modulus decreased by 14.6%, and hardness decreased by 30.7%), 6 weeks(modulus decreased by 31.5%, and hardness decreased by 46.3%), and 8 weeks(modulus decreased by 37.2%, and hardness decreased by 49.1%). In OVX group, significant differences of modulus between 4 weeks and 6 weeks, 4 weeks and 8 weeks were detected. And there were also significant differences of hardness between 4 weeks and 6 weeks, 4 weeks and 8 weeks in OVX group. No temporal changes in cortical bone of elastic modulus and hardness were detected in sham group.

### 4. FTIR

**Fig 5:**
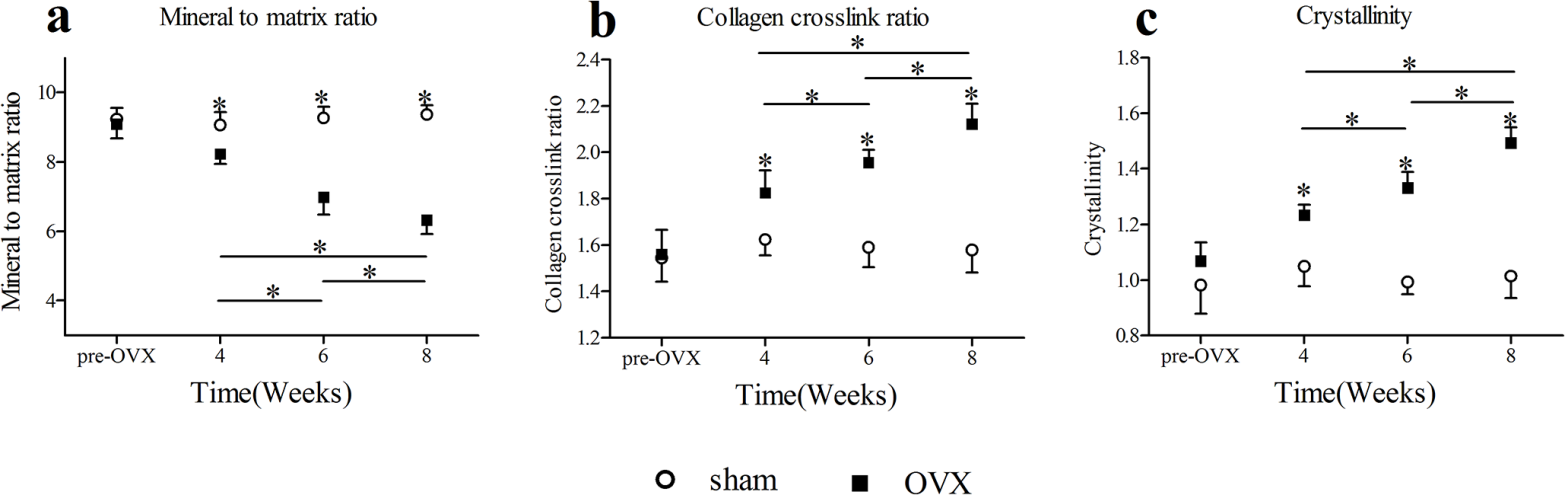
FTIR results(mean ± SD) of cortical bone specimens. Asterisks showed the statistically significant difference between OVX group and sham group at the same time point. 4 week onwards the values of (a)mineral to matrix ratio, (b)collagen crosslink ratio, and (c)crystallinity in OVX group decreased significantly. Transverse lines with asterisks above showed the differences in OVX group among different time points.*p < 0.05.

Results of FTIR analysis on femoral cortical bone specimens were shown in Fig 5. In cortical bone of OVX group, the mineral to matrix ratio decreased with time, while both collagen crosslink ratio and crystallinity increased with time. 4 weeks onwards the values of mineral to matrix ratio, collagen crosslink ratio, and crystallinity of cortical bone in OVX group changed significantly relative to sham group at 4 weeks(mineral to matrix ratio decreased by 9.3%, collagen crosslink ratio increased by 12.3%, crystallinity increased by 17.5%), 6 weeks(mineral to matrix ratio decreased by 24.6%, collagen crosslink ratio increased by 22.9%, crystallinity increased by 34.0%), and 8 weeks(mineral to matrix ratio decreased by 32.5%, collagen crosslink ratio increased by 34.4%, crystallinity increased by 47.2%). In OVX group, significant differences of mineral to matrix ratio between 4 weeks and 6 weeks, 6 weeks and 8 weeks, 4 weeks and 8 weeks were detected. There were significant differences of collagen crosslink ratio between 4 weeks and 6 weeks, 6 weeks and 8 weeks, 4 weeks and 8 weeks. Also, There were significant differences of crystallinity between 4 weeks and 6 weeks, 6 weeks and 8 weeks, 4 weeks and 8 weeks. No temporal changes in cortical bone of mineral to matrix ratio, collagen crosslink ratio, and crystallinity were detected in sham group.

### 5. Mechanical tests

**Fig 6:**
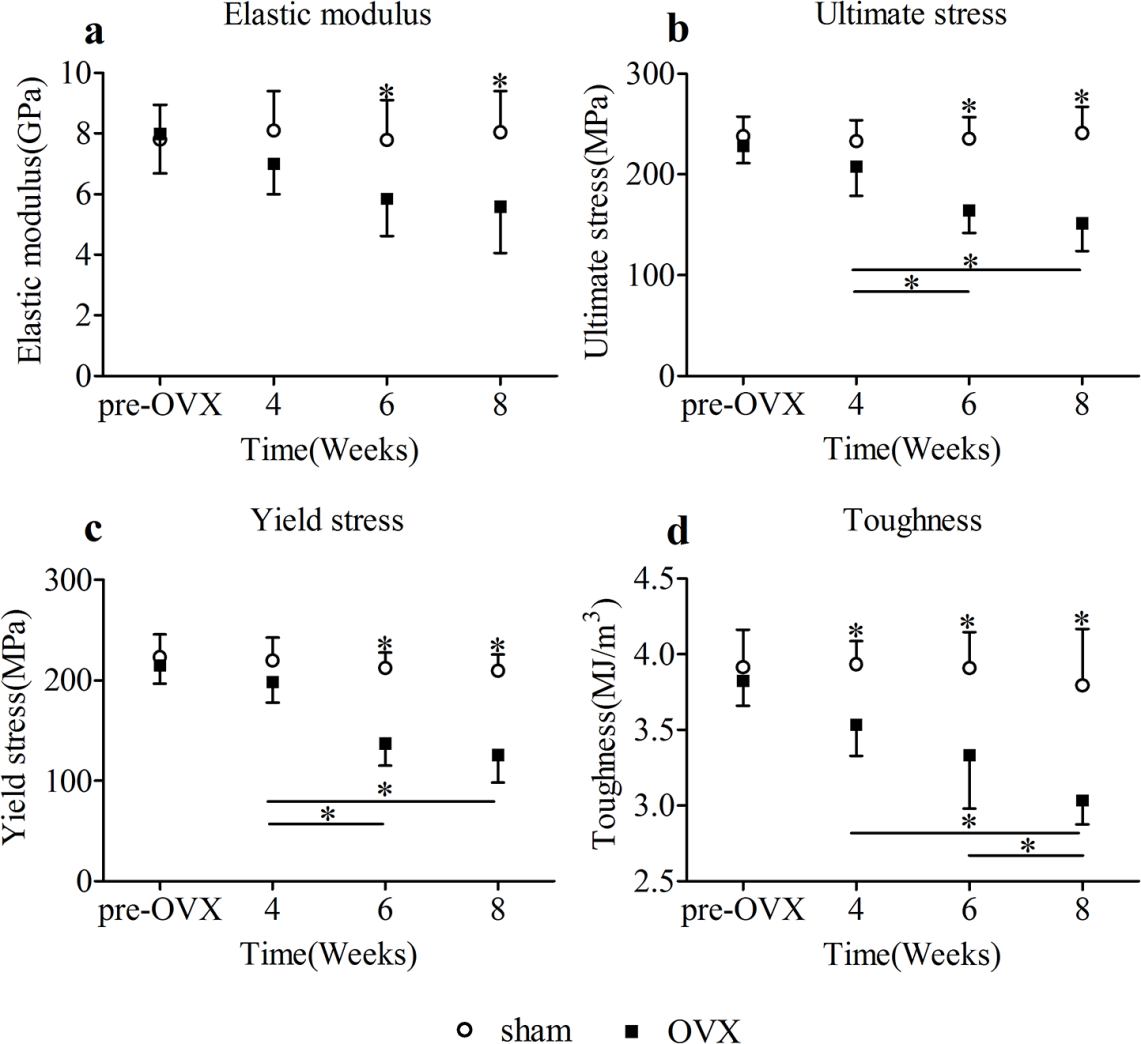
Biomechanical results(mean ± SD) of femoral cortical bone specimens. Asterisks showed the statistically significant difference between OVX group and sham group at the same time point. At 6 and 8 weeks after surgery in OVX group, the elastic modulus(a), ultimate stress(b), and yield stress(c) decreased significantly. At 4, 6, and 8 weeks after surgery in OVX group, the toughness(d) decreased significantly. Transverse lines with asterisks above showed the differences in OVX group among different time points. *p < 0.05.

Elastic modulus, ultimate stress, and yield stress strength of specimens in OVX group, measured using 3-point bending tests, decreased in terms of time(Fig 6). 6 weeks onwards, significant decline of elastic modulus, ultimate stress, and yield stress in OVX group was detected relative to sham group. 4 weeks onwards, significant decline of bone toughness in OVX group was detected relative to sham group. At 6 and 8 weeks after surgery in OVX group, the elastic modulus decreased by 24.8% and 30.5%, the ultimate stress decreased by 30.2% and 37.2%, the yield stress decreased by 35.4% and 39.9%, respectively, comparing to those in sham group. At 4, 6, and 8 weeks after surgery in OVX group, the bone toughness decreased by 10.2%, 14.7%, and 20.1%. In OVX group, significant differences of ultimate stress were observed between 4 weeks and 6 weeks, 4 weeks and 8 weeks. Also significant differences of yield stress were observed between 4 weeks and 6 weeks, 4 weeks and 8 weeks in OVX group. Significant differences of bone toughness were observed between 6 weeks and 8 weeks, 4 weeks and 8 weeks. Elastic modulus of cortical bone in OVX group also presented a gradual decrease with age, but the the differences among different time points were not statistically significant. No temporal changes of elastic modulus, ultimate stress, yield stress, and toughness were detected in sham group.

### 6. Relationships among BMD, structure, nanomechanical, FTIR, and biomechanical parameters

We summarized the R^2^ values among BMD, structure, nanomechanical, FTIR, and biomechanical parameters in sham group and OVX group separately(Tables 1 and 2). In sham group, no significant correlation among BMD, structure, nanomechanical, FTIR and macromechanical parameters were detected. Only the correlation between cortical thickness and ultimate stress was statistically significant, but the R^2^(R^2^ = 0.233) value was quite low.

**Table 1:** R^2^ values among BMD, structure, nanomechanical, FTIR, and biomechanical parameters studied in sham group.

R^2^(+/-)	BMD	Cortical thickness	Cortical area fraction	Modulus	Hardness	Mineral to matrix ratio	Crystallinity	Collagen cross-link ratio	Elastic modulus	Ultimate stress	Yield stress	Toughness
BMD	1	0.081(+)	0.006(-)	0.553(-)	0.0002(+)	0.072(+)	0.00001(-)	0.00003(+)	0.0001(+)	0.0007(-)	0.0001(-)	0.003(-)
Cortical thickness		1	0.008 (-)	0.012(-)	0.115(+)	0.040(-)	0.006(+)	0.024(+)	0.166(+)	0.233^a^(+)	0.166(+)	0.019(-)
Cortical area fraction			1	0.001(-)	0.058(+)	0.139(-)	0.0003(+)	0.0004(+)	0.027(-)	0.019(+)	0.022(+)	0.049(+)
Modulus				1	0.000004(+)	0.022(+)	0.024(+)	0.001(-)	0.045(+)	0.098(+)	0.096(+)	0.009(+)
Hardness					1	0.069(-)	0.006(-)	0.041(+)	0.011(-)	0.147(+)	0.111(+)	0.024(-)
Mineral to matrix ratio						1	0.005(+)	0.011(-)	0.002(-)	0.004(+)	0.013(-)	0.009(+)
Crystallinity							1	0.019(-)	0.194(+)	0.002(+)	0.031(+)	0.023(+)
Collagen cross-link ratio								1	0.001(-)	0.009(+)	0.021(+)	0.053(+)
Elastic modulus									1	0.153(+)	0.020(+)	0.0007(+)
Ultimate stress										1	0.419(+)	0.094(+)
Yield stress											1	0.022(+)
Toughness												1

Note: Modulus and hardness were the results of nanoindentation tests. Elastic modulus, ultimate stress, yield stress, and toughness were the results of 3-point bending tests. R^2^ values were presented in the table cells. (+) means positive correlation, and (-) means negative correlation. R^2^>0.75 was considered as strong, and 0.75>R^2^>0.50 was considered as moderate. R^2^ values lower than 0.5 were classified as parameters having weak relationship.

^a^P<0.05 was considered statistically significant.

**Table 2:** R^2^ values among BMD, microstructure, nanomechanical, FTIR, and biomechanical parameters studied in OVX group.

R^2^(+/-)	BMD	Cortical thickness	Cortical area fraction	Modulus	Hardness	Mineral to matrix ratio	Crystallinity	Collagen cross-link ratio	Elastic modulus	Ultimate stress	Yield stress	Toughness
BMD	1	0.001(+)	0.009(-)	0.053(+)	0.117(+)	0.069(+)	0.004(-)	0.067(-)	0.028(-)	0.048(+)	0.0002(-)	0.083(+)
Cortical thickness		1	0.003(+)	0.097(+)	0.016(+)	0.012(+)	0.016(-)	0.0004(-)	0.001(-)	0.013(+)	0.005(+)	0.032(+)
Cortical area fraction			1	0.035(+)	0.006(+)	0.097(+)	0.045(-)	0.030(-)	0.184(+)	0.00001(-)	0.0167(+)	0.049(+)
Modulus				1	0.765^a^(+)	0.838^a^(+)	0.729^a^(-)	0.773^a^(-)	0.357^a^(+)	0.666^a^(+)	0.681^a^(+)	0.601^a^(+)
Hardness					1	0.645^a^(+)	0.588^a^(-)	0.727^a^(-)	0.300^a^(+)	0.520^a^(+)	0.428^a^(+)	0.390^a^(+)
Mineral to matrix ratio						1	0.724^a^(-)	0.754^a^(-)	0.519^a^(+)	0.479^a^(+)	0.664^a^(+)	0.734^a^(+)
Crystallinity							1	0.803^a^(+)	0.382^a^(-)	0.580^a^(-)	0.675^a^(-)	0.526^a^(-)
Collagen cross-link ratio								1	0.345^a^(-)	0.594^a^(-)	0.563^a^(-)	0.466^a^(-)
Elastic modulus									1	0.082^a^(+)	0.314^a^(+)	0.371^a^(+)
Ultimate stress										1	0.715^a^(+)	0.418^a^(+)
Yield stress											1	0.535^a^(+)
Toughness												1
				

Note: Modulus and hardness were the results of nanoindentation tests. Elastic modulus, ultimate stress, yield stress, and toughness were the results of 3-point bending tests. R^2^ values were presented in the table cells. (+) means positive correlation, and (-) means negative correlation. R^2^>0.75 was considered as strong, and 0.75>R^2^>0.50 was considered as moderate. R^2^ values lower than 0.5 were classified as parameters having weak relationship.

^a^P<0.05 was considered statistically significant.

In OVX group, the correlations between BMD and macromechanical parameters were not statistically significant. The correlations between structural parameters(cortical thickness and cortical area fraction) and macromechanical parameters were not statistically significant. In OVX group, for nanomechanical parameters, mineral to matrix ratio, and collagen crosslink ratio had strong correlations with tissue modulus measured with nanoindentation(R^2^>0.75). Crystallinity had moderate correlation with tissue modulus measured with nanoindentation(R^2^>0.50). Mineral to matrix ratio, crystallinity, and collagen crosslink ratio had moderate correlation with hardness measured with nanoindentation(R^2^>0.50). For biomechanical parameters, crystallinity, and collagen crosslink ratio all had moderate correlations with ultimate stress(R^2^>0.50) and yield stress(R^2^>0.50), except that mineral to matrix ratio had a little weak correlation with ultimate stress(R^2^ = 0.479). Mineral to matrix ratio had a moderate correlation with elastic modulus measured with 3-point bending test. With regard to bone toughness, moderate correlation were detected between bone toughness and mineral to matrix ratio(R^2^>0.50), crystallinity(R^2^>0.50). In OVX group, evaluating the interrelationship between FTIR parameters themselves, mineral to matrix ratio correlated well to crystallinity and collagen crosslink ratio. Also, crystallinity correlated well to collagen crosslink ratio. Evaluating the interrelationship between nanomechanical parameters themselves, E correlated well to H. Evaluating the interrelationship between biomechanical parameters themselves, ultimate stress correlated well to yield stress, toughness correlated well to yield stress.

## Discussion

The estrogen withdrawal induced by OVX surgery, affected the mineral and collagen properties of cortical bone. And the alterations of mineral and collagen properties measured with FTIR correlated well with nanomechanical and whole-bone mechanical properties. However, estrogen withdrawal did not affect BMD and structure of cortical bone. This might indicate that, the decline in femoral midshaft strength might be attributed to the alterations of mineral and collagen properties measured with FTIR.

Characteristics of mineral crystal and collagen, nanomechanical and whole-bone macromechanical properties changed significantly in OVX group relative to sham group. The experimental results in OVX group showed time related changes in the mineral crystal and collagen, which corresponded to decrease in elastic modulus and hardness measured by nanoindentation, and decrease in whole bone strength as the duration of estrogen withdrawal increased. The moderate to strong correlation, between FTIR results and nanomechanics, also between FTIR results and macromechanics, indicated that the alterations of mineral crystals and collagen might affect the nanomechanical and macromechanical properties of cortical bone. BMD measured by DXA, and structure measured by μCT stayed constant in OVX group until the end of study. And this was consistent with the former study by Liu[25] in our department, even the time length of our study was 8 weeks, longer than Liu’s. The decline of femoral midshaft strength were not attributed to BMD and structure of cortical bone in our study.

Alterations of cortical bone mineral and collagen properties detected by FTIR and nanomechanics detected by nanoindentation in OVX group were significantly different from sham group in our study from 4 weeks on. While the alterations of whole-bone biomechanics, detected by 3-point bending test, were significant from 6 weeks on. The nanomechanical parameters, including elastic modulus and hardness, had strong correlations with bone mineral and matrix properties detected by FTIR. This indicated that the cortical bone tissue level mineral and matrix properties, nanomechanics changed earlier than whole-bone level macromechanics.

With regard to the nanomechanics of cortical bone in OVX group, significant reductions of tissue modulus and hardness were detected by nanoindentation in our study in 4 weeks. At the end of the experiment, modulus decreased by 37.2%, and hardness decreased by 49.1%. In cortical bone of OVX rats, significant reductions of modulus and hardness were also detected by nanoindentation 4 weeks after surgery[26]. Chang et al.[27] reported that in control and sham operated mice, the elastic modulus of tibia cortical bone was 26–30 Gpa, and the hardness was 0.9–1.1 Gpa. The elastic modulus of tibia cortical bone in OVX mice decreased to 15–20 Gpa, and hardness decreased to 0.3–0.7 Gpa. There was about a 37.5% decline in modulus, and a 50% decline in hardness of cortical bone. The decline percent was similar to our study. In our study, the tested sites of nanoindentation were only located on interstitial bone. The information about what happened in nanomechanical properties of osteonal bone was not reported. In the nanoindentation tests, dehydrated bone samples used in our study had the obvious limitations that the conditions of the specimens were not the same as the in vivo tissue condition. Although dehydration increases tissue modulus and hardness[28, 29], all samples were treated similarly, and therefore the comparisons of relative mechanical properties were relevant.

In the regression analysis within sham or OVX group, there were no statistically significant correlations between BMD and structural parameters. Also there were no statistically significant correlations between BMD and macromechanical parameters. These results were consistent with the former study on OVX rabbits[30]. This indicated that the deterioration of femoral midshaft bone strength in our study might not be attributed to BMD and structural parameters. In the regression analysis within sham group, the correlation between FTIR results and nanomechanical, macromechanical parameters were not statistically significant. However, in the regression analysis within OVX group, this correlation was statistically significant. This might indicate that estrogen withdrawal was primarily responsible for the changes of mineral and collagen properties. And the changes of material measured with FTIR was responsible for the changes of nanomechanical and macromechanical parameters.

Mineral to matrix ratio is representative of the amount of mineral normalized to the amount of collagen present. It is a measure of BMD and correlates with ash weight measurements, but its values are not comparable with BMD since it is a measure of mineral per amount of collagen present[31]. Bone apatite is a rigid material that can not dissipate much energy, and it has been widely suggested that the collagen matrix plays a key role in bone toughness[32]. The ratio of mineral to matrix could reflect hyper—or hypo mineralization that can deteriorate bone, because it encompasses both major constituents of bone(mineral and collagen). As bone is considered to be a composite material, and its mechanical properties depend on the quantity and quality of both mineral and collagen[31]. The decline of this ratio in our rabbit model was similar to human cortical bone[7]. Decreased mineral to matrix ratio in OVX group indicated the imbalance between mineral and collagen composition, and might cause the degradation of the bones’ mechanical properties[33]. This was verified by the strong correlation between mineral to matrix ratio and elastic modulus detected by nanoindentation. And this ratio also correlated with the whole bone-level strength, the decreased ratio of mineral to matrix ratio made contribution to the the decreased ultimate stress and yield stress in whole-bone strength. Both tissue level and whole-bone level mechanics correlated with the alteration of mineral to matrix ratio in this study. The strong correlation between mineral to matrix ratio and tissue level or whole bone-level mechanics was also observed in mouse[34] and rat[35].

Crystallinity is correlated with mineral crystal size and perfection as determined by X-ray diffraction line broadening[36]. A three-scale finite element investigation has demonstrated that mineral crystal size could be responsible for experimental nanoindentation results from cortical bone[6]. The earlier transmission electron microscopy (TEM) studies investigated by Baud[37] and Boskey[38] have reported that mineral crystal size increased with the onset of osteoporosis and the results here suggested that the crystal dimensions would contribute to changes in the tissue level response in osteoporotic bone. However, in another study on OVX ovine model, FTIR analysis found that crystallinity was significantly reduced following 12 and 31 months of estrogen deficiency. What called for special attention were that the animal model was different, and preparation methods of the bone specimens for FTIR analysis were quite different[39]. The increased crystal size could affect the crystallinity detected by FTIR[40]. The alteration of crystallinity in osteoporotic cortical bone contributed to the alteration of nanomechanics, and the increasing of crystallinity was concomitant with the duration of estrogen withdrawal in our study. The finding of an association between increased crystallinity and decreased bone strength was also in agreement with several other studies. Increased particle size was detected obtained from biopsies of patients that had femoral neck fracture compared with controls[41]. And increased crystal size was also found in biopsies from patients with osteoporosis compared with controls in FTIR analysis[13, 42] and Raman study[43]. There are several possible reasons that may explain the association between crystallinity and bone strength. First, the larger and more perfect bone mineral crystals may represent those that remain when bone is remodeled, and hence may indicate the increased tissue turnover and the loss of younger, newly formed bone tissue. The smaller crystals are generally more soluble and can be dissolved early and easily. Smaller crystals also indicate newly formed bone; hence, decreasing of smaller crystals means less formation and/or more resorption. Second, from physicochemical principles, larger particles generally tend to be more brittle and weaker. Because when a force is applied, the atoms generally try to move in relation to the adjacent layer of atoms. In metals, for example, making the particles smaller generally strengthens the materials. Broadening the size distribution also strengths the material[44]. Finally, larger crystals may not be able to align quite well with the collagen matrix, weakening the crystal-mineral interactions, and making the bone tissue weaker.

One of the most important molecular properties of collagen is its cross-link, which provides the fibrillar matrices with various mechanical properties such as tensile strength and viscoelasticity[45]. Collagen maturity is estimated by the 1660/1690 cm^−1^ subbands ratio of the amide I band. This ratio has been related with the degree of pyridinium(mature) relative to reducible (immature) types of collagen cross-links[22]. As the duration of estrogen withdrawal increased, the increasing of collagen cross-link ratio is concomitant with the deceasing of tissue-level and whole bone-level strength. The significant correlation between collagen cross-link ratio and elastic modulus or hardness indicated the nanomechanics was affected by the interaction among collagens. Also this correlation was detected between collagen cross-link ratio and whole bone strength, which indicated this influence could be amplified to whole-bone level.

The increase in collagen cross-link ratio in osteoporosis was detected in studies on human[9–11]. The collagen cross-link ratio was significantly higher in both IOP(idiopathic osteoporosis with idiopathic fractures) and ILBMD(idiopathic low BMD but no fractures) groups than healthy controls[46]. Increased collagen cross-link ratio was significantly associated with fracture risk. In ovariectomized monkey model, a decreased collagen cross-link ratio in the cortical bone region of tibia was detected[47]. But with regard to the trabecular bone in this study, an increased collagen cross-link ratio was detected. The author explained that the change in the organic component of bone due to ovariectomy is site-specific. The experimental period of this study was 2 years. In another study on ovariectomized monkey model, no significant difference of collagen cross-link ratio in cortical region of humerus was detected between OVX group and sham group[48]. The length of experimental time was 18 months. This might indicate that, OVX may have different effects on collagen cross-link among different sites. In an OVX ovine model, collagen cross-link was significantly increased 12 months after post-OVX and then reduced to control levels after 31 months[39]. This might indicate that the changes of collagen cross-link were time-related. The time length of observation in our study was 8 weeks. To assess the collagen cross-link state more thoroughly, a longer time length might be needed.

In our study, we observed an increase in collagen cross-link ratio in cortical bone of femoral midshaft, and this increase was associated with a decline in cortical bone strength. The increase in collagen cross-link ratio indicated a significant increase in non-reducible collagen cross-links. The proper collagen matrix is important for regulation of mineral deposition and is a major contributor to the strength of the composite tissue[49]. Increased collagen cross-linking may reflect the improper collagen matrix. The increased collagen cross-link ratio may be because of the possibility that the matrix produced in OVX matures at a faster rate than in normal bone matrix or the bone matrix of OVX undergoes post-translational modification for a longer period of time than the bone matrix of normal bone, perhaps because of a delay or alteration in mineralization. Meanwhile, the state of collagen cross-link ratio was only one of the many determinants of cortical bone strength. The collagen content and structure were also important for mechanical integrity. Detailed spectra processing shows that the amide I band is a composite of collagen molecular groups, water molecules, and non-collagenous proteins. Only the increased collagen cross-link ratio detected by FTIR might not reflect the whole collagen cross-link state.

With regard to DXA and structural properties, no significant changes were found between OVX and sham groups during the experiment. It might be attributed to that the time length of the experiment was not long enough to detect the BMD and microstructure in cortical bone on this osteoporotic rabbit. Numerous studies have documented that low bone density is significantly associated with the risk of osteoporotic fractures[50]. And structural properties such as cortical thickness, cortical cross-section area measured with μCT also contribute to bone’s mechanical competence[51]. However, in our study, the constant BMD, cortical thickness, and cortical area in OVX group might have only little effect on bone strength. The decreased cortical bone strength might be mainly attributed to the alterations of bone material characteristics as stated above. BMD, structure, bone mechanics on tissue-level and whole bone-level, and bone material properties stayed constant in sham group. This indicated that the cortical bone of intact rabbits in our experiment was mature and constant.

Rabbits are the species known to have quite fast Haversian bone remodeling processes. The pathognomonic issue in rabbits is that OVX alone might not be enough to develop low BMD despite estrogen deficiency[52]. Compared with former studies, our study demonstrated that estrogen withdrawal in OVX rabbits could have detrimental effects on cortical bone, and these detrimental effects were attributed to the alterations of mineral and collagen properties. The estrogen withdrawal did not affect cortical bone BMD and structure. On OVX rats, 7 weeks of estrogen withdrawal did not affect cortical bone BMD, and cortical thickness[53]. The cortical bone strength in that study did not change. But they used a mid-diaphyseal section(cortical bone only) in the compression study. The different macromechanical tests should be noticed. Another study with a duration of 10 weeks on OVX rats also found no changes of BMD in cortical bone of femoral midshaft[54]. Cross-sectional geometry and density of the femoral mid-diaphysis cortical bone measured with μCT did not detect significant differences between sham and OVX rats[55]. OVX may have more of an effect on the trabecular bone region than on the cortical bone region on BMD in the initial period following ovariectomy. There might be regional differences in the response to OVX. The region that was abundant of cancellous bone was more sensitive to estrogen withdrawal than cortical bone region.

## Conclusions

In conclusion, our study demonstrated the time related alterations of mineral and collagen properties, and mechanics on nano and whole-bone level in cortical bone of OVX rabbits. The alterations of crystal size and collagen maturity affected the nano and whole-bone level mechanics. As bone has a hierarchical architecture, this indicated that the alterations at material level could affect the microscale and whole bone-level mechanics.

## Supporting Information

S1 Table R^2^ values among BMD, microstructure, nanomechanical, FTIR, and biomechanical parameters studied in pooled data. (DOCX)
