# Supplementary material for: Time Related Changes of Mineral and Collagen and Their Roles in Cortical Bone Mechanics of Ovariectomized Rabbits
Source: PLoS One. 2015 Jun 5;10(6):e0127973. doi: 10.1371/journal.pone.0127973 (PMC4457815; doi:10.1371/journal.pone.0127973)
Supplement: S1 Table — (DOCX) [file pone.0127973.s001.docx]

# Supporting information

S1 Table R^2^ values among BMD, microstructure, nanomechanical, FTIR, and biomechanical parameters studied in pooled data.

| R^2^(+/-) | BMD | Cortical thickness | Cortical area fraction | Modulus | Hardness | Mineral to matrix ratio | Crystallinity | Collagen cross-link ratio | Elastic modulus | Ultimate stress | Yield stress | Toughness |
| --- | --- | --- | --- | --- | --- | --- | --- | --- | --- | --- | --- | --- |
| BMD | 1 | 0.024(+) | 0.060(-) | 0.043(+) | 0.080(+) | 0.086(+) | 0.029(-) | 0.061(-) | 0.0002(+) | 0.046(+) | 0.011(-) | 0.050(+) |
| Cortical thickness |  | 1 | 0.004(-) | 0.034(+) | 0.026(+) | 0.004(+) | 0.006(-) | 0.0001(-) | 0.021(+) | 0.040(+) | 0.022(+) | 0.006(+) |
| Cortical area fraction |  |  | 1 | 0.002(+) | 0.005(-) | 0.0007(+) | 0.000003(-) | 0.0003(-) | 0.002(+) | 0.002(-) | 0.004(+) | 0.009(-) |
| Modulus |  |  |  | 1 | 0.752^a^(+) | 0.859^a^(+) | 0.744^a^(-) | 0.780^a^(-) | 0.389^a^(+) | 0.714^a^(+) | 0.724^a^(+) | 0.604^a^(+) |
| Hardness |  |  |  |  | 1 | 0.675^a^(+) | 0.675^a^(-) | 0.668^a^(-) | 0.289^a^(+) | 0.638^a^(+) | 0.564^a^(+) | 0.432^a^(+) |
| Mineral to matrix ratio |  |  |  |  |  | 1 | 0.757^a^(-) | 0.778^a^(-) | 0.433^a^(+) | 0.591^a^(+) | 0.672^a^(+) | 0.666^a^(+) |
| Crystallinity |  |  |  |  |  |  | 1 | 0.734^a^(+) | 0.280^a^(-) | 0.607^a^(-) | 0.625^a^(-) | 0.513^a^(-) |
| Collagen cross-link ratio |  |  |  |  |  |  |  | 1 | 0.344^a^(-) | 0.596^a^(-) | 0.580^a^(-) | 0.460^a^(-) |
| Elastic modulus |  |  |  |  |  |  |  |  | 1 | 0.245^a^(+) | 0.336^a^(+) | 0.311^a^(+) |
| Ultimate stress |  |  |  |  |  |  |  |  |  | 1 | 0.766^a^(+) | 0.517^a^(+) |
| Yield stress |  |  |  |  |  |  |  |  |  |  | 1 | 0.535^a^(+) |
| Toughness |  |  |  |  |  |  |  |  |  |  |  | 1 |

Note: Modulus and hardness were the results of nanoindentation tests. Elastic modulus, ultimate stress, yield stress, and toughness were the results of 3-point bending tests. R^2^ values were presented in the table cells. (+) means positive correlation, and (-) means negative correlation. R^2^>0.75 was considered as strong correlation, and 0.75>R^2^>0.50 was considered as moderate correlation. R^2^ values lower than 0.5 were classified as parameters having weak relationship.

^a^P<0.05 was considered statistically significant.
